# Factors affecting patient satisfaction at a plastic surgery outpatient department at a tertiary centre in South Africa

**DOI:** 10.1186/s12913-023-10050-4

**Published:** 2023-09-29

**Authors:** Chrysis Sofianos

**Affiliations:** Division of Plastic and Reconstructive Surgery, University of the Witwatersrand, Johannesburg, South Africa

**Keywords:** Patient health, Patient satisfaction, Loyalty, Plastic surgery technique, Management, Batho Pele principles, Service quality, Organisation success, Patient experience

## Abstract

**Background:**

The main purpose of a medical facility is to improve the welfare of patients, and user satisfaction is one of its primary goals. This study aimed to identify variables influencing patient satisfaction at the Plastic Surgery Outpatient Department of Chris Hani Baragwanath Academic Hospital, Gauteng, South Africa. By identifying factors affecting patient satisfaction, the services provided to patients may be improved.

**Methods:**

A questionnaire was compiled focusing on patients over 18 years of age and using a Likert scale to measure factors influencing patients’ satisfaction with the services received. Descriptive statistics were applied.

**Results:**

A total of 142 participants, 69% of whom were under age 40 and 52.8% were male, were recruited using a systematic sampling technique. In 78% of cases, this was the patient’s first visit to the clinic. The results revealed that the patients were not satisfied with the ease of appointment scheduling and waiting times. A high level of satisfaction was reported for both nursing staff and doctors, aside from a lack of clear and prominent display of name badges. Overall satisfaction was at the “satisfied” level, and no participants reported lower than neutral feelings. The CSAT score was 79.5%, which is considered “good” for healthcare. The participant’s level of education was significant in multiple items; those with the lowest education reported higher overall satisfaction. A model produced by regression analysis was found to be strongly significant statistically.

**Discussion:**

Batho Pele principles in South Africa provide a framework for consulting with service consumers, ascertaining their happiness, and identifying methods to enhance their experience. According to this survey, people are happy with the human part of the services received, although areas such as scheduling appointments and the physical surroundings still need much work.

**Conclusion:**

These aspects of treatment are occasionally disregarded in a medical organisation with insufficient finances. Developing these areas can help turn patients into devoted patrons of a healthcare facility. An effective strategy to boost customer satisfaction has been suggested to enhance service quality and, especially in South Africa, conform to Batho Pele service standards.

## Introduction

Sub-Saharan Africa is unique in that decades of financial constraints and various health system pressures have resulted in patient dissatisfaction, poor quality of care, underutilisation, and various barriers to access to such an extent that service users cannot imagine a health establishment functioning in any other way [1].

Batho Pele, a seSotho phrase meaning “people first,” was launched in 1997 and instituted as a framework to transform public services at all levels to improve service delivery [2]. Batho Pele aims to change the attitudes of public servants, so they are committed to service delivery and shift from a rules-based approach to a results-driven approach that fosters innovation [2]. The slogan of Batho Pele summarises the key beliefs of the approach—“we belong, we care, we serve.” [2].

Strategic health management (SHM) identifies the concrete steps that must be taken to realise the goals of patient-centred and value-based medicine [3]. In the SHM system, an emerging approach is the retrospective study of organisational events to direct future actions. Business process management (BPM) is an approach that aims to continuously improve service delivery by analysing key operations in line with the strategies of an organisation through a well-designed, implemented, integrated, executed, and monitored management approach [4].

The healthcare industry is growing rapidly, and recently, there has been a shift from outcomes-based to systems-based approaches in performance management [4]. The primary goal of a health facility is to promote patient wellbeing [5]. The top priorities for a healthcare organisation are patient experience and patient satisfaction [6]. The healthcare industry is turning towards the business world for practices and principles that aim to achieve an ideal trade-off between achieving efficiency and promoting patient wellbeing and responsiveness [4].

A key component of BPM is benchmarking, and determining patient satisfaction with current services is the first step [7]. Providers and healthcare organisations can obtain useful insights, including the effectiveness of care provided, by measuring patient satisfaction [6]. By analysing patient satisfaction, areas needing improvement can be identified to optimise quality of care and direct interventions instituted by healthcare managers and ultimately move toward a BPM approach to providing outpatient services [6]. Business process re-engineering and benchmarking facilitate significant improvements in patient satisfaction [4].

Stanton, in his seminal definition of a service in 1974, described it as a grouping of intangible activities that are separately identifiable and provide satisfaction when marketed to a consumer [8]. A service is an essentially intangible act offered by one party to another and does not result in any ownership thereof [9]. Intangibility remains an essential component to defining a service, because services are actions rather than specific objects and cannot be seen, tasted, felt, or touched as are tangible products [8]. A service also remains inseparable from the person or company providing it [9]. The difficulties inherent in this are that services cannot be readily displayed, easily communicated, inventoried, or patented legally, and the true costs of a “unit of service” are difficult to determine [10].

Today, the patient is a customer who buys or obtains health services [11]. Patients, as consumers, have certain rights and entitlement to the delivery of quality healthcare [11]. Healthcare organisations have shifted towards functioning as a service industry, including the employment of human resources (HR) professionals and graduates of management programs [12].

Consumers expect to share in critical decisions, secure help when required, and be able to actively dictate, devise, and realise their own consumer journey [13]. The historical understanding of “patients” as people in pain suffering in silence and wholly dependent on healthcare professionals has evolved to the modern “patients-as-consumers” definition of efficient, experience-elevated, independent, and technologically savvy consumers of healthcare services [13].

Customer satisfaction is defined as the percentage of overall customers who report an experience with an organisation and its products or services as exceeding specified goals of satisfaction [14]. This essentially translates to how well an organisation’s service, products, and overall customer experience are able to meet customers’ expectations [15]. Customer satisfaction is generally described in terms of “an evaluative, affective, or emotional response.” [16].

The increase in competition has prompted many organisations to seek ways to differentiate themselves, and a key strategy resulting in success is the delivery of high quality services and increasing customer satisfaction [14]. A 2006 ground-breaking study by Fornell and colleagues at the University of Michigan illustrated the strong relationship between customer satisfaction and fiscal success by creating a hedge portfolio in which stocks were bought long and sold short based on changes in the American Customer Satisfaction Index (ACSI) [17]. Changes in customer attitudes align with noticeable differences in quality of service, and changes in customer satisfaction indices do not happen overnight [18].

The dimensions of service quality, namely reliability, assurance, tangibles, empathy, and responsiveness, were defined in 1988, and the result is known as the SERVQUAL scale [19]. SERVQUAL, as an assessment tool, has been used extensively in literature assessing quality of healthcare services [20]. This scale is based on the concept of the disconfirmation paradigm. The dimensions are quantifiable by “obtaining measures of expectations and perceptions of performance levels of service attributes relevant to each dimension, calculating the difference between expectations and perceptions of actual performance on these attributes, then averaging excess attributes.” [19].

Each dimension may be further understood as follows [19]:

- Tangibles – physical facilities, equipment, and appearance of personnel.
- Reliability – the ability of the organisation to perform the promised service accurately and dependably.
- Responsiveness – willingness to help the customer and provide prompt service.
- Assurance – employee courtesy, knowledge, and ability to inspire trust and confidence.
- Empathy – the individualised caring and attention paid to customers.

Theoretical models of customer satisfaction provide valuable insights into the determinants of satisfaction [21]. Research in customer satisfaction identifies four models of customer satisfaction—the expectancy–disconfirmation model (EDM), the performance model, the rational expectations model, and the expectations artefact model [22].

Marketing studies have applied EDM to predict repurchase intentions, consumer preferences, and customer loyalty [23]. The relevance of EDM to this study is that it has become the predominant model to explain citizen satisfaction with a public service [24]. Furthermore, its relevance is clear, as it is a useful model to conceptualise patient satisfaction [23]. EDM focuses on the psychology of citizens and parallels the development of “behavioural public administration” in which psychology enhances the practices and outcomes of public administration [24]. The model comprises four distinct but interrelated components—expectations, performance, disconfirmation, and satisfaction [24]. Expectations refer to a customer’s anticipation of the performance of a service; perceived performance looks at the client experience after using a service or product and may exceed or fall short of the expectations they had [24]. Disconfirmation may be understood as the gap between customer expectations and actual performance, and is divided into three types—positive, negative, and simple disconfirmation [25]. If actual product or service performance cannot meet a customer’s expectations, negative disconfirmation occurs leading to poor satisfaction while if the reverse is true, positive disconfirmation occurs leading to good customer satisfaction [25]. Simple disconfirmation is when there is no difference between actual performance and customer expectations of a product or service [25]. The relevance of this model and diagram is that good patient satisfaction ultimately results from positive disconfirmation, in that the service patients receive at the outpatient department exceeds their expectations of that service. In South Africa, the limited availability of healthcare facilities means that although patients have negative disconfirmation, they are forced to use the same service, as no alternative is available.

When a patient lives in a first-world, or high-income, country, the probability of being satisfied with the country’s healthcare system is 3,400 times higher than that of patients in low-income countries [26]. Wealthier countries are better able to keep patients satisfied, largely because of higher public spending on healthcare [26].

The literature highlights that patient expectations and perceptions of service quality both vary in different contexts and that demographic variables influence these perceptions and expectations disparately [27]. Documented links exist between expectations and perceptions of service quality in relation to certain demographics, such as previous use of a specific healthcare service, individual values, lifestyle, inpatient vs outpatient, level of education, and frequency of visits [27].

Patients of different ages have varying expectations of healthcare service, with age an important factor highlighted in numerous studies [28]. Healthcare professionals should therefore take age and the concomitant differences in expectations into account when providing healthcare services [28].

Previous studies have highlighted that patient satisfaction with their healthcare experience includes many influencing factors, such as medical expenses, service attitude, medical facilities, visiting environment, and others [28].

Healthcare organisations are complex adaptive systems that have the primary aim of providing quality healthcare to the citizens of a country. In South Africa, Batho Pele informs all public service interactions and puts people first. The Constitution of South Africa ensures that all citizens have timeous access to comprehensive and equitable healthcare of sufficient quality. Principles of service quality, customer satisfaction, variables affecting the patients’ experiences, and their ultimate satisfaction have been explored and form the basis of this research. Quality improvement should form the cornerstone of any modern healthcare organisation, and data gleaned from studies into the factors affecting patient satisfaction should inform future improvement initiatives.

## Research contextual setting, aim, and objectives

Chris Hani Baragwanath Academic Hospital (CHBAH), in Gauteng Province, South Africa, manages approximately 592,000 outpatients every year [29]. The fire at Charlotte Maxeke Johannesburg Academic Hospital (CMJAH) 20 kms away, forcing closure of parts of that hospital, has increased the number of patients attending facilities of CHBAH [29]. In 2018, South Africa had only 148 plastic surgeons registered with the Association of Plastic, Reconstructive, and Aesthetic Surgeons of South Africa (APRASSA) with this number remaining relatively stable since then [30]. With CHBAH serving as the tertiary referral centre for large parts of the country, the number of patients in the Plastic Surgery Outpatient Department (PSD) is significant [29]. It is well known that long waiting times result in lower patient satisfaction [31]. By identifying other factors that influence patient satisfaction, a reorganisation or improvement of the experience in the PSD may be realised. The importance to CHBAH of such a study is that adopting a strategic plan aimed at improving patient satisfaction would lead to improved services provided to end users, despite the lack of resources and growing numbers of patients attending its facilities. South Africa is on the road to adopting a National Health Insurance (NHI), and such a setup would require constant benchmarking and adoption of strategic plans to provide and manage cost-effective healthcare services with patient satisfaction as an important indicator of effective functioning of such a health service. There is a paucity of research on patient satisfaction in South Africa, and more specifically in outpatient settings, and this study provides valuable insight within this third-world context.

The study aimed to investigate outpatients’ perceptions of the factors affecting the services provided and their effect on patient satisfaction at the Plastic Surgery Department of Chris Hani Baragwanath Academic Hospital, Gauteng. The objectives were:

1. To identify demographic factors influencing patient satisfaction with an outpatient plastic surgery service.
2. To determine patient satisfaction with various components of an outpatient plastic surgery service.
3. To determine overall patient satisfaction with an outpatient plastic surgery service.
4. To determine whether a predictive model of overall patient satisfaction with an outpatient service can be developed.
5. To provide recommendations for improving patient satisfaction as part of a strategic healthcare plan and incorporate the Batho Pele principles of quality service delivery.

## Methods

### Research design

The data collection instrument was a self-administered questionnaire in English, which was prepared based on the literature review. A self-administered questionnaire was selected because it has the important advantage of ensuring patient anonymity, thus facilitating more valid and truthful responses [32]. SERVQUAL, as described in the literature review, was used as the basis to construct the questionnaire [19]. The questionnaire was divided into two parts. The first part collected patient demographic data and referral information, without any personally identifiable information. The second part focused on aspects of outpatient visits that are believed to affect patient satisfaction. This was subdivided into six topics—physical setup and environment, appointment scheduling and waiting times, nursing staff, doctors, communication, and overall satisfaction—as suggested in the literature. The second part used closed answers on a five-point Likert scale for all satisfaction-related questions (1 – very satisfied, 2 – satisfied, 3 – neutral, 4 – dissatisfied, and 5 – very dissatisfied). The five points on the Likert scale were combined into a three-scale structure for easier descriptive interpretation by combining “satisfied” and “very satisfied” as satisfaction, and “very dissatisfied” and “dissatisfied” as dissatisfaction. The Likert scale is considered fundamental and is the most frequently used tool for assessing consumers’ attitudes toward a particular product or service [33].

### Target population and sampling

Patients under the age of 18 are considered a vulnerable population in research because of their limited intellectual and emotional capacity, and obtaining proper informed consent is difficult [34]. The target population for the study therefore included patients above the age of 18, both new and follow-up, presenting to the PSD of CHBAH, Gauteng.

To attend the clinic, patients must have a referral, that is, they cannot self-present for consultation. Referrals are either internal, from other outpatient clinics in the hospital, or external. There is only one PSD at this hospital, with an outpatient clinic held every Monday starting at 7 a.m. The target population (N) is approximately 120 patients seen monthly at the PSD of CHBAH.

Target sample size (n) was determined using the single population proportion formula [35] considering a patient satisfaction rate of 84% (P) in Gauteng Province [36], 5% sampling error (d), and a 95% confidence interval (Zα/2). Therefore, the calculated minimum sample size, assuming a nonresponse rate of 10%, was found to be 77.

The sampling strategy employed in this study was systematic sampling [37]. This is a probability sampling method, in which researchers select a member of the population at regular intervals (k) determined in advance. A list of the population is not available in advance. The population order is random; therefore, a representative sample is expected using the systematic sampling method, allowing for conclusions to be made about the population [37]. Systematic sampling remains the preferred method over simple random sampling when the risk of data manipulation is low and the relevant data do not show patterns [38]. There were no discernible patterns in the research population of this study.

The first patient was chosen using the lottery method. The interval (k) is calculated as the target population divided by the target sample size – N/n (120/77 = 1.55); therefore, every second patient was selected. Applying this approach, the number of respondents obtained was 142 patients, which exceeded the target population and sample size. The high number of respondents could be explained by the higher than average number of patients attending the clinic at the beginning of the year, as no outpatient clinics were held during December of the previous year.

### Pilot study

A pilot study is an important component of a research project and is used to test research protocols, the research instrument, and the sample recruitment approach [39]. Before embarking on the actual data collection for the study, the questionnaire was piloted on 12 patients in the Outpatient Department. All patients found the questionnaire to be clear, easily understood, and had no problem answering the questions. They also confirmed that the participant information sheet was easy to follow, so no changes were made to either instrument. The data from the pilot study were not included in the overall research sample.

### Administration of questionnaire

The questionnaire was administered over four consecutive Monday outpatient clinics at the PSD of CHBAH in January and February 2023. A quiet, private room with comfortable seating was available to the study participants to complete the questionnaire. Having multi-lingual availability for a questionnaire increases the chance of comprehensive, reliable, and fully representative data [40].

Patients with a low literacy level or non English speaking were able to request assistance in English, Afrikaans, isiZulu, seSotho, or seTswana, with assistance provided by staff at the PSD. No patients spoke a language other than those previously mentioned.

### Data analysis, validity, and reliability

The data were collected using the questionnaire described above. No identifying data were recorded on the questionnaire, and a unique study number was assigned to each respondent. The five-point Likert scale scores of satisfaction for each respondent were added, and the mean of the sum was determined to ascertain factors affecting satisfaction. The satisfaction score was divided into two categories—dissatisfaction (greater than 30) and satisfaction (less than or equal to 30). Data were then processed in Microsoft Excel, version 16, for Mac (Microsoft Corporation, Redmond, USA). The data were analysed using descriptive statistics, and the results included frequencies, mean ± standard deviation, median, and range. Data were then entered and analysed in SPSS (SPSS Statistical Package Version 23, IBM, Chicago, USA). The Shapiro–Wilk test of normality was used to check whether data were normally distributed, and a statistical test was then selected. *P* < 0.05 was considered significant.

The content and construct validity of the questionnaire was determined after review and discussion with a clinical researcher and biostatistician [41]. Face validity of the questionnaire was determined in discussion with the respondents of the pilot study. Multiple studies have been performed on patient satisfaction in various hospital departments and have been used as the gold standard to determine criterion-related validity. Higher population validity is likely to be the case in this research, as patients attending the PSD of CHBAH, Gauteng Province, had varied demographic characteristics in terms of gender, education status, and socioeconomic status.

To ensure reliability, the collected questionnaires were reviewed after every collection period (once a week) and checked for clarity, completeness, consistency, and accuracy of data.

### Considerations of bias

Social desirability bias is the tendency to respond in a way that pleases those performing the research [42]. Performing the interview on hospital premises increased the risk of social desirability bias, as would be the case if a health professional administered the questionnaire. The design of the study as a self-reported questionnaire and allowing patients to drop responses in a box at the exit of the department decreased the risk of social desirability bias. Dissent bias, the opposite of acquiescence bias, causes respondents to disagree with all statements presented [43]. Neutral response and dissent bias were minimised by explaining clearly the impact and usefulness of the study in the information sheet. There was a potential risk of cognitive bias in patients who had attended other outpatient departments or hospitals and may have had negative experiences that left them with predetermined views. The questionnaire used in this research urged them to answer based on their interaction and satisfaction with the specific PSD.

To avoid coercion bias, patients were provided with an information sheet and questionnaire upon arrival at the department. The usual waiting time is approximately one hour, so this gave them adequate time to consider participating in the study. It was stressed to them that participation was completely voluntary and would not affect their treatment. Completing the questionnaire occurred only after consultation with the healthcare professional.

## Results

The results of this study will be presented and discussed in five parts. First, the demographics of the sample will be described for the 142 participants in the survey. Second, descriptive statistics for the 45 items eliciting responses as to their satisfaction with the services and facilities at the PSD of CHBAH will be presented, and whether each item deviated above or below the overall mean satisfaction response of the others. Third, differences in means between pairs of subgroups within each demographic variable will be tested for significance on all 45 satisfaction items, and any significant results will be presented. Fourth, any significant correlations between the age variable and the ratings on the 45 satisfaction items will be reported. The final part will report the results of the effort to find a predictive model of overall satisfaction with the service received at the PSD.

### Sample demographics

The literature review highlighted that patient expectations and perceptions of service quality and satisfaction are influenced unevenly by demographic variables [27]. Furthermore, specific patient factors affecting perception of quality of a service are noted, such as gender, age, and education level [44]. Data on four demographic variables were collected during this research—age, gender, education level, and employment status.

Age data were collected as a direct report rather than by age range. Accordingly, it is treated as a continuous variable for descriptive and analytical purposes. The descriptive statistics are reported in Table 1. As the table reveals, the mean is appreciably higher than the median, indicating a pronounced degree of positive skewness. This degree of skewness was statistically significant at *p* = 0.0035 (2-tailed). Sixty-nine percent of the sample was under 40, and the remainder of the sample was between 40 and 62.

**Table 1:** Descriptive statistics for sample age distribution

N	Minimum	Maximum	Median	Mean	Std. Dev	Skewness	Std. Error of Skewness	Kurtosis	Std. Error of Kurtosis
142	19	62	33	36.04	12.35	0.592	0.203	–0.773	0.404

Day et al. looked at Health and Related Indicators in the South African Health Review and stated that South Africa is undergoing a demographic transition; the elderly population is growing [45]. The demographics from this research show that most patients attending the PSD are under 40; however, the older age groups should not be discounted as their population is growing, as mentioned above. Trauma accounts for a large proportion of presentations to plastic surgeons in South Africa, and with mostly younger individuals afflicted by trauma, this accounts for the positive skewness of the age of participants in this research.

Table 2 presents the category distributions for the three remaining demographic variables. Note that for gender, the transgender category contains only three people. This is not a large enough group to be included in statistical analyses, and consequently will be excluded from all gender analyses. However, the aim is not to discount the importance of providing adequate and equitable healthcare services to transgender patients. In South Africa, transgender patients have been faced with substandard and discriminatory healthcare provision [46].

**Table 2:** Frequency distributions for gender, education level, and employment status

Gender			Education Level			Employment Status		
Category	Frequency	Percent	Category	Frequency	Percent	Category	Frequency	Percent
Female	64	45.1	Did Not Attend	12	8.5	No	101	71.1
Male	75	52.8	Primary School	24	16.9	Yes	41	28.9
Transgender	3	2.1	High School	83	58.5	Total	142	100
Total	142	100	University	23	16.2			
			Total	142	100			

Table 2 reveals that the sample was close to evenly divided between males and females, that high school was the predominant level of education of participants, and that over 70% of the participants were not currently employed.

In addition to demographic questions, participants were asked to respond to four questions relating to the precursors and logistics of their visit to the PSD. The PSD does not accept self referrals; over 92% of referrals to the PSD were from a local clinic. More than 70% of patients used a taxi as the mode of transportation to the hospital. The importance of collecting this data is echoed by findings that effective coordination of non-urgent transportation for patients improves the user experience and reduces no-show rates to 4% [47].

### Descriptive statistics for the 45 satisfaction items

Satisfaction with the consultation room conditions was much higher than with those of the waiting area. The waiting area at the PSD usually has up to a hundred patients waiting at any one time, and with such high volumes, it is expected that noise levels are high, and seating and lighting are sometimes inadequate. There was an unusually wide range of satisfaction regarding the level of noise in the waiting area. Apparently, some patients find that a noisy environment reduces their level of satisfaction, while others are not bothered by it.

The literature highlights that social behaviour influences sound levels and that some patients prefer talking to others rather than enforcing a “no-talking” rule [48]. The Cultural Atlas states that most South Africans have a direct style of communication, and those with an Afrikaans or Black heritage (particularly females) tend to speak louder than their counterparts from the Western English-speaking world [49]. South Africans may also become uncomfortable with prolonged silence and naturally converse to fill it [49]. This may account for the varied perceptions of noise in the PSD. Only regarding cleanliness were the ratings of both the consultation room and waiting area close to equal. Despite the high numbers, it is positive that cleanliness is maintained in both of these areas.

Except for “waiting time for doctor,” the means of all other items under “appointment and scheduling” were below the midpoint of the scale, indicating that the various aspects addressed under this topic were not well received by patients. The PSD, at present, does take bookings; however, it does not work on a time slot basis, and usually patients do not have a choice of dates for appointments but rather as availability dictates. Satisfaction with ease of scheduling appointments is low. Satisfaction with waiting time until the appointment date as well as entering the PSD upon arrival are low. Lack of flexibility in appointments and long waiting times adversely affect patient satisfaction, [31] and this was confirmed by this study. This means that CHBAH should investigate methods to improve waiting times. The current appointment booking system is manual and entered in a book; a digital system could improve flexibility and availability of appointments, leading to increased patient satisfaction [31].

Satisfaction with the nursing staff had mean ratings of approximately 4.00 on the 5-point scale on ten of the eleven items under this topic, which represents a very high degree of satisfaction. Nurses are the backbone of any healthcare system, and the COVID-19 pandemic highlighted that the severe shortage of nurses will only worsen unless definitive steps are taken to improve the situation [50]. Despite resource constraints, and high work volumes, the nursing staff at the PSD were able to deliver care that resulted in good patient satisfaction. The only item that deviated appreciably from the high level of satisfaction with nursing care at the PSD pertained to the issue of whether name badges were displayed prominently. Clear and prominent name badges improve communication and ultimately patient satisfaction [51]. CHBAH does not provide name badges to its employees at present. Employees must purchase their own name badges. This research highlights the importance of the visibility of a name badge for patients attending services at a healthcare organisation. CHBAH and the Gauteng Department of Health could benefit from standardised, easy-to-read name badges provided to all employees, and guidelines could be developed to make name badge display mandatory to achieve the Batho Pele principles as outlined above.

The means of the ratings received with regard to satisfaction with doctors were approximately 4.00 on the 5-point scale for nine of the eleven items under this topic, which again represents a high degree of satisfaction. On the item pertaining to whether their name badges were displayed prominently, doctors were consistent with the nurses in deviating appreciably from the high satisfaction ratings they received on 82% of the items.

The only other item on which a degree of dissatisfaction was expressed pertained to the availability of a sufficient number of doctors to provide the level of service the patients desired. This, of course, is not a factor within the control of the doctors, but rather an indication of inadequate staffing levels. The chairperson of the South African Medical Association, Dr Mvuyisi Mzukwa, sounded the alarm in late 2022 that the staff shortage of healthcare workers in South Africa had hit a critical level, with a widening of the ratio to 0.8 doctors per 1000 patients [52]. Too few healthcare workers available to provide a service ultimately filters down to inadequate service provision and poor patient satisfaction. These research findings demand action from the local all the way to the national level in addressing the current staff shortage in the public health service in South Africa.

Mean satisfaction with communication with patients just barely edged into the “satisfied” zone of the scale and was consistent at this level among all six items under this topic. Communication, in combination with physical environment, customer friendly environment, privacy and safety, and responsiveness, is a key factor affecting healthcare service quality, subsequent patient satisfaction, and ultimately patient loyalty [53]. Patients are more loyal to a healthcare organisation if they are satisfied with the service received [53]. The importance of communication for patient satisfaction, as identified above, means that the results of this study—barely achieving “satisfaction”—is not good enough for a healthcare organisation that is attempting to improve the service it provides the citizens of Gauteng Province. A communication plan could be developed, and staff training could be initiated to improve this outcome.

The mean overall satisfaction level is virtually exactly at the “satisfied” level, which is halfway between neutral and very satisfied. No participants reported feeling less than neutral in their overall satisfaction with PSD, but 36.6% rated their overall satisfaction as neutral, 34.5% were highly satisfied, and 28.9% rated it as just satisfied. When looking at the literature, the overall satisfaction level of 79% shown in this study is comparable to other outpatient departments in third-world countries [54].

Considering customer satisfaction metrics, the customer satisfaction score is a widely-used metric to analyse overall customer satisfaction [55]. The overall CSAT in this study was 79.5%; the benchmark for CSAT in healthcare is 79% [55]. The CSAT score in this research study closely mirrors what is considered “good” for the healthcare industry; however, many factors have been highlighted that can be improved, resulting in a better score.

### Pairwise differences between demographic subgroups on satisfaction items

With the objective of identifying demographic variables significant to patient satisfaction, the subgroups within each of the four demographic variables were compared on their ratings of the satisfaction items to identify any disparities, indicating that certain subgroups perceive themselves as being underserved by certain aspects of the PSD and the services it provides. The results of these analyses of the satisfaction items will be presented serially for each demographic variable. Only items on which significant pairwise differences occurred will be reported.

#### Gender

The genders differed in their mean satisfaction ratings on physical setup and environment. Males were significantly more satisfied than females with the signage and directions to the PSD and with the consultation room lighting. The results of these differences are presented in Table 3.

**Table 3:** Items on which the Genders Differed Significantly in Satisfaction with the PSD’s Physical Setup and Environment

Item #	Gender Means		*t*-test Results	
	Male	Female	*t*	*p*
Signage/directions to PSD	3.47	3.06	2.622	.01
Consultation room lighting	3.69	3.44	2.548	.012

Previous research has shown that males tend to report higher customer satisfaction in general, but also specifically in healthcare services (patient satisfaction), compared to their female counterparts [56]. In their research, Otani et al. showed significance in the relationship between gender and nursing care, physician care, and overall helpfulness [57]. Male patients tended to rate their relationship with the doctor as more important to overall patient satisfaction, while females value the relationship with nurses more highly [57]. Overall helpfulness during the experience was more important to patient satisfaction for females [57].

This research did not echo the gender differences observed with overall satisfaction, nursing staff, doctors, and communication, and there were no further significant differences between the genders in their satisfaction ratings on the items pertaining to the other four specific topics addressed by the survey (i.e., appointment scheduling / waiting time, nursing staff, doctors, and communication) or on the overall satisfaction item.

#### Education Level

The evidence concerning the effects of education level on perception of healthcare and satisfaction thereof is varied and multiple. In the 1990s, a meta-analysis showed that patients with a lower education level tended to exaggerate the quality of services received and stated an overall higher level of patient satisfaction [58]. Other recent individual studies, however, fail to show significance [59].

The dominant pattern of differences when considering education level was a significantly higher satisfaction among those with the lowest education levels compared with all three higher education levels, especially regarding the waiting room environment, ease of scheduling, and waiting times. These are illustrated in Tables 4 and 5.

**Table 4:** Items on which education levels differed significantly in satisfaction with the PSD’s Physical Setup and Environment

Item Number	Education Level Means				*p*-values of Significant Post Hoc Pairwise Differences			
	(1) Did Not Attend	(2) Primary School	(3) High School	(4) University	(1): (2)	(1): (3)	(1): (4)	(3): (4)
Signage/directions to PSD	4.08	3.29	3.18	3.26	-	.046	-	-
Waiting area cleanliness	4.17	2.79	3.28	3.04	.001	.018	.004	-
Waiting area noise	4.25	2.50	2.58	2.78	< .001	< .001	.001	-
Waiting area seating	3.75	2.38	2.55	2.65	.001	.003	.005	-
Waiting area lighting	4.17	2.63	2.54	2.57	< .001	< .001	< .001	-
Consultation room cleanliness	3.83	2.88	3.14	3.13	.017	-	-	-
Consultation room noise	3.83	3.08	3.40	2.83	-	-	.005	.036
Consultation room seating	4.25	3.46	3.60	3.52	.046	-	-	-
Consultation room lighting	3.75	3.46	3.66	3.26	-	-	-	.005

**Table 5:** Items on which education levels differed significantly in satisfaction with appointment scheduling and waiting time at the PSD

Item Number	Education Level Means				*p*-values of Significant Post Hoc Differences		
	(1) Did Not Attend	(2) Primary School	(3) High School	(4) University	(1): (2)	(1): (3)	(2): (3)
Ease of scheduling	2.00	2.54	2.71	2.74	-	.038	-
Appointment reminder	1.42	1.92	2.13	1.83	-	.003	-
Waiting time for appointment	2.25	2.67	2.42	2.61	-	-	.031
Waiting time for doctor	2.92	3.87	3.82	3.52	.023	.025	-

Of the 66 pairwise comparisons between the four education levels on the 11 items under satisfaction with staff, seven reached statistical significance when looking at nursing staff, and six when looking at doctors. These significantly differing pairwise comparisons are reported in Tables 6 and 7. Participants at the lowest education level had significantly higher satisfaction ratings on two of the three items on which significant pairwise differences occurred, accounting for six of the seven such significant differences in nursing staff and accounting for all six of the significant differences with regard to doctors.

**Table 6:** Items on which education levels differed significantly in satisfaction with the nursing staff at the PSD

Item Number	Education Level Means				*p*-values of Significant Post Hoc Differences			
	(1) Did Not Attend	(2) Primary School	(3) High School	(4) University	(1): (2)	(1): (3)	(1): (4)	(2): (4)
Friendliness of nursing staff	4.25	3.71	3.95	4.35	-	-	-	.049
Nursing staff available to help	4.08	2.87	2.98	3.09	.002	.002	.007	-
Nursing staff name display	4.17	2.58	2.51	2.52	< .001	< .001	< .001	-

**Table 7:** Items on which education levels differed significantly in satisfaction with doctors at the PSD

Item Number	Education Level Means				*p*-values of Significant Post Hoc Differences		
	(1) Did Not Attend	(2) Primary School	(3) High School	(4) University	(1): (2)	(1): (3)	(1): (4)
Doctors' availability to help	3.67	2.33	2.60	2.65	.003	.008	.029
Doctors' display of their name	3.50	2.58	2.35	2.39	.027	< .001	.002

There were no significant pairwise differences between education levels in mean satisfaction ratings on any items under the communication topic or in overall satisfaction. As shown above, this study highlighted that patients with lower education levels had higher satisfaction ratings in several areas. This is consistent with the meta-analysis referred to earlier and supports the significance of education level in affecting patient satisfaction [58]. It could account for the good CSAT score and acceptable overall patient satisfaction score achieved in this research.

#### Employment status

In their study, Aloh et al. showed that employment status was statistically significant in determining overall patient satisfaction, patient experience, and rating of quality of care in an ambulatory health service in Nigeria [60].

None of the comparisons between the two categories of employment status on the 44 items subsumed under the five topics in this study reached statistical significance, nor did the means of the two employment status categories on the overall satisfaction item.

#### Age

It has been shown that doctors tended to have more patient-centred encounters with elderly patients, resulting in higher patient satisfaction with increasing age [61]. In the study of an ambulatory health service in Nigeria referred to above, age was found to be the strongest predictor of overall patient satisfaction [60].

Satisfaction items within only two of the topic categories manifested significant correlations with age. Within the physical setup and environment topic, nine of the ten satisfaction items correlated significantly with age; in the communications topic, four of the six items correlated significantly with age. Table 8 presents all these significant correlations.

**Table 8:** Satisfaction items manifesting significant correlations with age

Item Number	Item	Correlation	*p*
3.1	Signage/directions to PSD	.292***	< .001
3.2	Waiting area cleanliness	.226**	.007
3.3	Waiting area noise	.195*	.020
3.4	Waiting area seating	.263**	.002
3.5	Waiting area lighting	.319***	< .001
3.6	Consultation room cleanliness	.241**	.004
3.7	Consultation room noise	.245**	.003
3.8	Consultation room seating	.184*	.029
3.10	Consultation room privacy	.191*	.023
7.1	Nurses' communication ability	.208*	.013
7.2	Doctors' communication ability	.204*	.015
7.4	Explaining treatment	.265**	.001
7.6	Giving understandable explanations	.276**	.001

^*^p ≤ .05

^**^p < .01

^***^p < .001

### Predictive modelling of overall satisfaction

Regression analysis is an important statistical approach for determining the relationship between several factors and outcomes in healthcare [62].

An effort was made to ascertain whether a regression model could be developed that would account for a useful proportion of variance in overall satisfaction. Given the five topics under which the satisfaction items were organised, an attempt was made to determine whether any subset of the items associated with each of the topics could be combined to form a reliable scale for that topic. Items within each topic were subjected to principal components analysis to identify which items were loaded on the first component, and then the components with eigenvalues > 1.0 were rotated via the varimax method to identify the unique components. Alpha reliabilities were computed on the full set of each topic’s items, on the items loading on the first unrotated component, and on the items loading at > 0.40 on each of the rotated factors. The highest that any of these reliabilities reached for any of the topics was 0.611, which is substantially below the minimum required for an adequate multi-item scale (conventionally, 0.75 when used for research purposes only). Consequently, regression model building was conducted on the individual items.

A backward elimination strategy of variable retention in the model was used to identify the optimal subset of variables. This strategy tends to achieve higher coefficients of determination than forward selection or stepwise strategies. This resulted in an optimal model consisting of 16 variables. Table 9 presents a summary of the model’s efficacy, and Table 10 presents the coefficients and their *p*-values for each of the items included in the model.

**Table 9:** Summary of the model’s efficacy in explaining the variance of overall satisfaction

R	R Square	Adjusted R Square	SE of the Estimate	*F* (16,125)	*p*
.532	.283	.191	.761	3.078	< .001

**Table 10:** Satisfaction items included in the model predicting overall satisfaction

Item Number	Item	Unstandardised Coefficients		t	*p*
		B	Std. Error		
(Constant)	(Constant)	4.321	1.476	2.928	.004
3.8	Consultation room seating	–.210	.109	–1.923	.057
3.9	Consultation room lighting	–.140	.116	–1.204	.231
3.10	Consultation room privacy	–.336	.111	–3.023	.003
4.4	Waiting time for appointment	.122	.122	.999	.320
4.5	Waiting time for PSD entry	.122	.116	1.048	.297
4.6	Waiting time for doctor	–.144	.088	–1.638	.104
5.1	Friendliness of nursing staff	.141	.079	1.794	.075
5.3	Nursing staff available to help	–.270	.079	–3.444	.001
5.5	Nursing staff empathy	.147	.075	1.953	.053
5.7	Nursing staff willingness to assist	–.092	.085	–1.081	.282
5.11	Nursing staff approachability	.120	.082	1.452	.149
6.2	Service received from doctors	.104	.083	1.251	.213
6.6	Doctors' knowledge	.104	.084	1.236	.219
6.10	Respect shown by doctors	.183	.086	2.139	.034
7.2	Doctors' communication ability	–.152	.102	–1.496	.137
7.6	Giving understandable explanations	.164	.109	1.501	.136

The model explained 28.3% of the variance in overall satisfaction in the sample, which in future samples is likely to drop to 19.1%. This proportion of variance explained is statistically significant at *p* < 0.001. Cohen’s ƒ2 index of effect size for regression has a value of 0.332, which is at the upper end of a medium effect size, approaching the threshold of 0.35 for a large effect size. This can be considered a useful level of prediction.

Table 10 reveals that the satisfaction items included in the model represented all five of the topical categories in the survey. Note that the coefficients for most items in the model did not reach statistical significance. This is a common occurrence with the use of the backward elimination strategy. This occurs because this method of variable selection often allows predictors to be retained that are not significant, but which are necessary for other variables to make a significant contribution to the explanation of the dependent variable’s variance. The most important result is that the model as a whole is strongly statistically significant.

## Discussion

The Gap Model of Service quality states that service quality is the gap between expected and perceived service [63]. In today’s age, patients have become consumers who buy or obtain health services, [11] and there is an expectation of receiving high quality service that will leave the patient satisfied and ultimately loyal to a healthcare organisation [15]. Service quality is the precursor to overall customer satisfaction, [14] which is a highly personal and variable assessment that ultimately affects an organisation’s profitability [64].

It has been suggested that the expectancy–disconfirmation model is the primary model explaining citizen satisfaction with a public service [24] and that an inverse relationship exists between expectations and patient satisfaction; as expectations increase, satisfaction declines [24]. The landmark SERVQUAL scale developed by Parasuraman is based on the disconfirmation paradigm and highlights the dimensions of service quality as reliability, assurance, tangibles, empathy, and responsiveness [19].

The objective of the primary research—to determine factors affecting patient perceptions and satisfaction—is fulfilled with the finding from the literature review that they are many, and evidence is varied in support of their relative importance. A clear link between perceptions of service quality in relation to demographics, such as lifestyle, individual values, and level of education, has been documented [27].

Forty-five satisfaction items were included in the questionnaire and were grouped into six topics—physical setup and environment, appointment scheduling and waiting time, nursing staff, doctors, communication, and an overall satisfaction score. The division into these categories, and the utilisation of multiple satisfaction items, adequately fulfils the objective of identifying specific factors affecting patient satisfaction.

Batho Pele sets the backdrop for consultation with the end users of a service, determining their satisfaction thereof, and finding ways to improve their experience. This study has shown that patients are satisfied with the human element of the service provided; however, components such as appointment setup and the physical environment require improvement. In a healthcare organisation with limited resources, these elements of the service are often overlooked. Improvement in these areas is useful for converting patients into loyal customers of a healthcare organisation. A strategic plan to augment patient satisfaction is suggested to improve the quality of service provided, and specifically in South Africa, to adhere to the Batho Pele principles of service delivery.

Factors affecting patient perception are multiple and vary in their significance. This agrees with the literature review [65] and supports the multifactorial view that should be taken when benchmarking in this area and developing strategies to improve patient satisfaction as part of SHM [66].

This research assessed multiple demographic factors and their role in influencing patient satisfaction. Male patients, those with a lower level of education, and patients who were older all rated their satisfaction levels higher in multiple areas of analysis. This agrees with the literature [28]. Employment status was found to be insignificant, in contrast to findings in the literature [60].

Overall satisfaction scored at the “satisfied” level, which is halfway between “neutral” and “very satisfied,” and no participants reported feeling less than neutral in their overall satisfaction with the PSD in this study. This echoes findings in a similar study in an outpatient dermatology department [67].

Sixteen items from the overall 45 items in the questionnaire were identified as significant to include in the predictive model that was successfully developed and found to be strongly significant statistically.

Quality healthcare care is that which is safe and effective, while providing a positive experience through a responsive and person-centred approach, [68] and quality improvement is a coordinated and systematic approach to solving problems affecting healthcare delivery [68]. This study sheds some light on factors that could be improved with an aim to providing quality healthcare to citizens utilising public healthcare services in outpatient departments, both in South Africa and in the rest of the world.

Organisations that adopt strategic organisational approaches to patient experience are usually more successful at removing barriers to patient-centred care and improving patient satisfaction [69].

### Recommendations

- A strategic plan is necessary for a health organisation to improve the quality of the service it provides, and specifically in South Africa, to adhere to the Batho Pele principles of service delivery.
- Benchmarking of patient satisfaction is an important component in BPM and SHM and informs the development of an outpatient department’s strategic outlook.
- Recognition that patient satisfaction is a multi-factorial process that is informed by demographics, setting, and its importance in creating loyal patients that return for future treatments at a healthcare facility.
- Cleanliness and hygiene in the physical environment are sometimes difficult to maintain in high-volume patient areas but should not be overlooked as a source of patient satisfaction with their healthcare encounter.
- Although older patients and patients with lower levels of education may perceive higher levels of satisfaction, their lower expectations and understanding should be recognised and a fair and equitable healthcare service provided.
- Prominent and clear display of name badges is lacking, and with its importance emphasised in the literature, should form an important aspect of establishing communication in public health establishments in South Africa, even to the extent of replacing these badges with badges like the bright yellow background and black text utilised in the UK NHS.

### Limitations of the current study and areas of future research

This research was limited to a single outpatient department at a very busy tertiary academic hospital in Gauteng Province, South Africa. Plastic Surgery Outpatient departments across the major hospitals in South Africa and the rest of the world have different setups, both physical and staffing, and generalisability of the study to those departments might be difficult. Plastic surgery outpatient departments rarely see urgent or emergency cases, and the demographics of the patients attending this department are different from those attending other outpatient departments. Chris Hani Baragwanath Academic Hospital is the largest tertiary referral centre in South Africa and has the largest plastic surgery department in the country. Other smaller plastic surgery departments or hospitals might have different patient profiles and areas where patient satisfaction differs. The research did not look at satisfaction with Plastic Surgery care and procedures specifically, but rather at the outpatient department itself.

This lays the basis for further study at a broader level, encompassing patient satisfaction in different specialties but also outpatient departments at different levels of hospitals across South Africa. Expanding the model to identify other areas of patient satisfaction could form the basis for future research. Once a strategic plan has been developed to improve patient satisfaction in the South African outpatient context, revisiting this study to see whether improvement has been realised could allow for the evaluation of the effectiveness of such an intervention. With NHI on the horizon, this study serves as an indicator of the status quo of patient satisfaction and what changes need to be instituted to ensure patient satisfaction once this universal healthcare program has been implemented.

## Data Availability

Ethical approval was contigent on all study data being stored electronically on an encrypted storage disk. The datasets used and/or analysed during the current study are available from the corresponding author on reasonable request.
